# Supplementary material for: A pH-driven transition of the cytoplasm from a fluid- to a solid-like state promotes entry into dormancy
Source: eLife. 2016 Mar 22;5:e09347. doi: 10.7554/eLife.09347 (PMC4850707; doi:10.7554/eLife.09347)
Supplement: Supplementary file 1. — DOI: http://dx.doi.org/10.7554/eLife.09347.039 [file elife-09347-supp1.docx]

**Table S1: *S. cerevisiae* strains used in this study**

| **Name** | **Genotype** | **Source** |
| --- | --- | --- |
| BY 4741, WT | Mat a, his3∆1, leu2∆0, met15∆0, ura3∆0, [RNQ^+^] |  |
| W 303, Ade^+^, WT | Mat alpha, can1-100, his3-11,15, leu2-3,112, trp1-1, ura3-1 | Gift, Zachariae lab |
| W 303, Ade^+^;  trp::GPD-ypHluorin2 | Mat alpha, can1-100, his3-11,15, leu2-3,112, ura3-1 | This study |
| W 303, Ade^+^;  trp::GPD-mCherry-GFP | Mat alpha, can1-100, his3-11,15, leu2-3,112, ura3-1 | This study |
| W 303, Ade^+^;  pAG415GPD-EGFP-µNS | Mat alpha, can1-100, his3-11,15, leu2-3,112, trp1-1, ura3-1; pAG415GPD-EGFP-µNS | This study |
| Acs1-GFP | BY 4741 | Huh et al. 2003 |
| Ade12-GFP | BY 4741 | Huh et al. 2003 |
| Ade17-GFP | BY 4741 | Huh et al. 2003 |
| Ade5-GFP | BY 4741 | Huh et al. 2003 |
| Ade7-GFP | BY 4741 | Huh et al. 2003 |
| Adh2-GFP | BY 4741 | Huh et al. 2003 |
| Ala1-GFP | BY 4741 | Huh et al. 2003 |
| Arc1-GFP | BY 4741 | Huh et al. 2003 |
| Asc82-GFP | BY 4741 | Huh et al. 2003 |
| Cdc19-GFP | BY 4741 | Huh et al. 2003 |
| Cdc60-GFP | BY 4741 | Huh et al. 2003 |
| Cpr6-GFP | BY 4741 | Huh et al. 2003 |
| Cys4-GFP | BY 4741 | Huh et al. 2003 |
| Dug2-GFP | BY 4741 | Huh et al. 2003 |
| Edc3-GFP | BY 4741 | Huh et al. 2003 |
| Erg6-GFP | BY 4741 | Huh et al. 2003 |
| Gcd2-GFP | BY 4741 | Huh et al. 2003 |
| Gcd6-GFP | BY 4741 | Huh et al. 2003 |
| Gcd7-GFP | BY 4741 | Huh et al. 2003 |
| Gcn3-GFP | BY 4741 | Huh et al. 2003 |
| Gdb1-GFP | BY 4741 | Huh et al. 2003 |
| Gln1-GFP | BY 4741 | Huh et al. 2003 |
| Gln4-GFP | BY 4741 | Huh et al. 2003 |
| Glt1-GFP | BY 4741 | Huh et al. 2003 |
| Gly1-GFP | BY 4741 | Huh et al. 2003 |
| Gph1-GFP | BY 4741 | Huh et al. 2003 |
| Gsy2-GFP | BY 4741 | Huh et al. 2003 |
| Hek2-GFP | BY 4741 | Huh et al. 2003 |
| Hem2-GFP | BY 4741 | Huh et al. 2003 |
| His4-GFP | BY 4741 | Huh et al. 2003 |
| Hsp104-GFP | BY 4741 | Huh et al. 2003 |
| Hsp42-GFP | BY 4741 | Huh et al. 2003 |
| Hsp82-GFP | BY 4741 | Huh et al. 2003 |
| Hts1-GFP | BY 4741 | Huh et al. 2003 |
| Ils1-GFP | BY 4741 | Huh et al. 2003 |
| Ira1-GFP | BY 4741 | Huh et al. 2003 |
| Kic1-GFP | BY 4741 | Huh et al. 2003 |
| Nop56-GFP | BY 4741 | Huh et al. 2003 |
| Pab1-GFP | BY 4741 | Huh et al. 2003 |
| Pex3-GFP | BY 4741 | Huh et al. 2003 |
| Pre6-GFP | BY 4741 | Huh et al. 2003 |
| Prs4-GFP | BY 4741 | Huh et al. 2003 |
| Psa1-GFP | BY 4741 | Huh et al. 2003 |
| Rim20-GFP | BY 4741 | Huh et al. 2003 |
| Rnr2-GFP | BY 4741 | Huh et al. 2003 |
| Rnr4-GFP | BY 4741 | Huh et al. 2003 |
| Rpl4B-GFP | BY 4741 | Huh et al. 2003 |
| Rpn9-GFP | BY 4741 | Huh et al. 2003 |
| Rps11B-GFP | BY 4741 | Huh et al. 2003 |
| Sam1-GFP | BY 4741 | Huh et al. 2003 |
| Sam2-GFP | BY 4741 | Huh et al. 2003 |
| Sbp1-GFP | BY 4741 | Huh et al. 2003 |
| Sgt2-GFP | BY 4741 | Huh et al. 2003 |
| Sis1-GFP | BY 4741 | Huh et al. 2003 |
| Snf7-GFP | BY 4741 | Huh et al. 2003 |
| Spc42-GFP | BY 4741 | Huh et al. 2003 |
| Ssa1-GFP | BY 4741 | Huh et al. 2003 |
| Ssa2-GFP | BY 4741 | Huh et al. 2003 |
| Ssb1-GFP | BY 4741 | Huh et al. 2003 |
| Ssb2-GFP | BY 4741 | Huh et al. 2003 |
| Ssd1-GFP | BY 4741 | Huh et al. 2003 |
| Sse1-GFP | BY 4741 | Huh et al. 2003 |
| Sse2-GFP | BY 4741 | Huh et al. 2003 |
| Sti1-GFP | BY 4741 | Huh et al. 2003 |
| Sui2-GFP | BY 4741 | Huh et al. 2003 |
| Thr1-GFP | BY 4741 | Huh et al. 2003 |
| Ths1-GFP | BY 4741 | Huh et al. 2003 |
| Tps2-GFP | BY 4741 | Huh et al. 2003 |
| Uga1-GFP | BY 4741 | Huh et al. 2003 |
| Ugp1-GFP | BY 4741 | Huh et al. 2003 |
| Ugt51-GFP | BY 4741 | Huh et al. 2003 |
| Ura2-GFP | BY 4741 | Huh et al. 2003 |
| Ura7-GFP | BY 4741 | Huh et al. 2003 |
| Ura8-GFP | BY 4741 | Huh et al. 2003 |
| Vas1-GFP | BY 4741 | Huh et al. 2003 |
| YAR009C-GFP | BY 4741 | Huh et al. 2003 |
| YLR143W-GFP | BY 4741 | Huh et al. 2003 |
| YMR253C-GFP | BY 4741 | Huh et al. 2003 |
