# Supplementary material for: A pH-driven transition of the cytoplasm from a fluid- to a solid-like state promotes entry into dormancy
Source: eLife. 2016 Mar 22;5:e09347. doi: 10.7554/eLife.09347 (PMC4850707; doi:10.7554/eLife.09347)
Supplement: Supplementary file 2. — DOI: http://dx.doi.org/10.7554/eLife.09347.040 [file elife-09347-supp2.docx]

**Table 2: Plasmids used in this study**

| **Name** | **Alternative Name** | **Source** |
| --- | --- | --- |
| pENTRY-µNS-stop | Y-521 | This study |
| pAG415GPD-EGFP-µNS | O-3185 | This study |
| pENTRY-ypHluorin2-stop | Y-717 | This study |
| pAG304GPD-ypHluorin2 | O-3192 | This study |
| pDUAL2HFG-µNS-sfGFP | O-3295 | This study |
| pDM353-µNS-GFP | O-3377 | This study |
