# Supplementary material for: A pH-driven transition of the cytoplasm from a fluid- to a solid-like state promotes entry into dormancy
Source: eLife. 2016 Mar 22;5:e09347. doi: 10.7554/eLife.09347 (PMC4850707; doi:10.7554/eLife.09347)
Supplement: Supplementary file 3. — DOI: http://dx.doi.org/10.7554/eLife.09347.041 [file elife-09347-supp3.docx]

**Table S3: Osmolality of media and buffers**

| **Medium*/ buffer*** | **Osmolality *(mOsm/kg)*** ± SD |
| --- | --- |
| *Standard 290 mOsm* | 308.5 ± 14.5 |
| *Standard 1000 mOsm* | 918.7 ± 8.0 |
| SD complete w/o g*lucose* | 216.3 ± 2.5 |
| SD complete 2% g*lucose* | 290.7 ± 4.7 |
| P buffer pH 5.5 2% g*lucose* | 296.7 ± 14.3 |
| *P buffer pH* 6.0 2% g*lucose* | 346.0 ± 2.6 |
| P buffer pH 7.4 2% g*lucose* | 381.3 ± 19.2 |
| P buffer pH 6.0, 1.0 M s*orbitol* | 1253.3 ± 5.5 |
